# Pragmatic clustered randomised control trial to evaluate a self-regulated learning intervention to be implemented in South Australian primary schools—study protocol

**DOI:** 10.1186/s13063-025-08814-w

**Published:** 2025-04-03

**Authors:** S.A Brinkman, B. Lam, S. Dawson, R. Marrone, D. Schunk, K. Winkel, H. Hermes, F. Gabriel, S. Fowler, D. Engelhardt

**Affiliations:** 1 Education Futures, University of South Australia, Adelaide, South Australia Australia; 2 Public and Behavioural Economics, Johannes Gutenberg University Mainz, Mainz, Germany; 3 Mathematics Education, University of Koblenz, Koblenz, Germany; 4 Ludwig Erhard Ifo Center for Social Market Economy and Institutional Economics, Ifo Institute, Fürth, Germany; 5 Department for Education, Adelaide, South Australia Australia

**Keywords:** Self-regulation, Metacognition, Pragmatic, Clustered, RCT, Primary school

## Abstract

**Background:**

Self-regulated learning (SRL) is described as a process whereby learners actively take control of their learning by setting goals, planning, monitoring, evaluating, and adjusting their learning strategies to improve performance and achieve desired outcomes Panadero (Front Psychol 8:422, 2017). SRL proficiency has been shown to predict educational success and lifelong outcomes, such as income and health. While SRL is recognised as a key lifelong competency, there remain questions regarding how educators can best develop and promote SRL in school settings. A scalable, low-cost intervention targeted at grade 1 students (6–7 years old) in Germany was found to have substantial effects on impulse control and self-regulated learning, with sustained impacts on long-term academic success Schunk (Nat Hum Behav 6(12):1680-90, 2022). This study protocol seeks to adapt the Schunk et al. (2022) randomised trial to the Australian content and extend it to grades levels 2, 4 and 6.

**Methods:**

We will use a standard pragmatic clustered (by school) randomised controlled superiority trial with an additional population-wide matched parallel control arm. Effectively, we will conduct three trials—one for each age/grade level. Each trial will be powered to assess the impact of the intervention on the age/grade groups independently: grade 2 (early primary, 7–8 years), grade 4 (mid primary, 9–10 years), and grade 6 (late primary, 11–12 years). Schools assigned to the treatment group will have all three grade levels (grades 2, 4, and 6) receiving the treatment (at least one class per grade); no classes in the schools assigned to the control group will receive the intervention. A minimum of 56 schools with an average cluster size of 19 children/class will be required to detect a minimum impact of 0.25 SD effect size at 80% power taking into account the clustered design with an intraclass correlation coefficient (ICC) of 0.05. This results in a total sample of 1064 per grade and thus 3192 students in total (56 schools per arm × 19 students in an average-sized class × three grade levels). Randomisation will occur on a 1:1 ratio, such that half of the schools (*n* = 28), and effectively about half of the students (*n* = 1596) will receive the intervention. The primary outcome will be improved self-regulation assessed at 6 weeks, 6 months and 12 months post the intervention. Longer-term secondary outcomes will include academic and wellbeing measures obtained through administrative data linkage to the National Assessment Program in Literacy and Numeracy (NAPLAN) outcomes and the Wellbeing and Engagement Collection (WEC) outcomes measures in the year following implementation (grades 3, 5, and 7). Follow-up via the South Australia Data Linkage Systems will allow for longer-term academic outcomes, mental health, school completion, criminal justice, and tax data.

**Discussion:**

This protocol paper provides a detailed record of the trial design. We also discuss our analytical plan, especially highlighting the opportunities associated with the linkage of the trial participants to South Australia’s population-wide administrative data linkage systems.

**Trial registration:**

ACTRN12623001331628. Registered on 9th of November 2023.

## Introduction

### Background and rationale {6a}

The terms metacognition, self-regulation, and self-regulated learning appear frequently in the educational literature and are often used in closely related contexts. Metacognition is described as the developmental aspects of how one monitors or thinks about one’s own cognition [1] or ‘thinking about thinking’ [2]. Self-regulation (SR) refers to the capacity to regulate attention, emotion, impulses, and behaviour directed at pursuing individually valued goals. The term self-regulated learning (SRL) is the application of metacognition and self-regulation in the context of education and learning settings.

An ever-growing body of research demonstrates that a child's early SRL plays a crucial role in their school readiness, school achievement, and in a range of later life outcomes [3–5]. Alternatively, poor SRL has been linked to low academic motivation, engagement, and achievement [6]. Studies undertaken in English speaking schools suggest that children experiencing risk, particularly those experiencing cumulative risk (e.g. being low-income and having English as a second language), are an important population for support interventions that seek to develop student SRL proficiency [7, 8]. Further, some research has demonstrated that disadvantaged children benefit the most from early SRL interventions [9] and as such, may be seen to be an effective policy investment to enhance equity.

The number of comprehensive intervention studies that target SRL in primary school aged students is growing. However, to date, these remain largely domain-specific, and the transferability of these skills across school subjects is unclear. For example, the association between SRL and academic performance seems to be particularly strong in the case of mathematics (see [10–15]). Similarly, research on the impact of SRL interventions on students’ reading and literacy comprehension also shows positive effects [3, 16]. In the Australian context, Harding [17] examined student SRL in maths and reading in grades 5, 6, 7, and 8 in 42 Victorian schools [17] demonstrating that higher-performing students regulate their learning to a higher proficiency than their lower-performing peers (utilising a self-report assessment of SRL).

A systematic review and meta-analysis published in 2018 highlights that SRL strategies can positively impact primary and secondary school children without requiring a heavy burden on teacher or student time and should be embedded into teaching practices [18]. The review demonstrated that SRL mindfulness-based interventions even of a short duration (6 months or less) had significant and lasting impacts on adolescent SRL [18]. For instance, children participating in a Head Start program in the US benefitted most from a short classroom-based intervention [14]. The authors suggested shorter interventions can have long-lasting impacts and influence student success. Studies noted in the review support the proposition that short-term interventions can be beneficial; however, the research has been largely limited to one age group. It is unclear if similar long-term effects are found across multiple ages and student populations.

In a meta-analysis by Xu et al. [19], the relationship between SRL and academic achievement in K-12 for online and blended educational contexts was examined. Their analysis supported the findings of an earlier meta-analysis by Dignath and Büttner [20], which looked at the effects of SRL interventions on academic performance, strategy use, and student motivation based on 49 primary-level and 35 secondary-level studies. Both analyses indicated that interventions had a higher effect size for primary school students than secondary students in terms of academic performance. For primary students, social cognitive theory-based programmes showed greater effect sizes than motivation or metacognition strategies. However, Dent and Koenka [21] noted that the strongest correlation between metacognitive strategies and academic outcomes was found among early primary students (K to grade 2), which weakened in later primary years (grades 3 to 5). The authors suggested that the weakening of effects may be due to the assessment methods used.

It is clear that whilst there is a growing body of research on SRL, important knowledge gaps remain. For instance, it is unclear if SRL skills transfer from one subject to another, or if they are domain-specific skills. Further, there are very few studies that examine multiple student populations conjointly and employ consistent outcome measures. Randomised control trials are rare, especially with long-term follow-up across a broad socio-economic spectrum. This study protocol presents a trial which aims to build on and somewhat replicate that of Schunk et.al. The Schunk et.al. study represents a scalable intervention that has shown a moderate to high impact on later academic outcomes. Conducted in Germany, the study demonstrated that 5 h of SRL training delivered over 5 weeks significantly impacted grade 1 student’s impulse control and self-regulation behaviour in the classroom. The authors also showed lasting improvements in academic skills including reading and identifying careless mistakes, which were targeted by their SRL intervention.

This trial will determine the impact and acceptability of the SRL intervention including the experiences and practical considerations of participants in the classroom. The training and implementation of the intervention in the school setting will build momentum within the education system and the findings of the trial provide robust evidence for scale-up. The trial will contribute to the academic literature in the following unique ways: it will determine the impact of a short-term scalable SRL intervention on multiple outcomes (self regulated learning skills, literacy, numeracy, self-esteem, resilience, perseverance, emotional regulation, positive relationships with others, student engagement with learning, and attendance) and for multiple grade levels within primary school. Further, the trial is powered to determine if the intervention enhances equity by lifting those students from disadvantaged backgrounds with greater magnitude. Lastly, too few studies are replicated in science, and this trial provides an opportunity to determine if we can reproduce the effects found by Schunk et al. (2022) within an Australian setting.

### Objectives {7}

1. Adapt the SRL intervention as utilised by Schunk et al. to the Australian context and to different age groups to allow for pragmatic implementation at scale within the South Australian public education system.
2. Investigate the effects of the SRL intervention by different grade levels and by socio-economic position.
3. Provide novel insights into the mechanisms underlying a potentially positive treatment effect.
4. Evaluate results and cost-efficacy of the intervention for potential scale-up within South Australia.

#### Specific hypotheses

1. We hypothesise the Australian adaption of the SRL intervention will increase primary school children’s self-regulated learning skills as measured by teacher ratings of self-regulated learning 6 weeks, 6 months and 12 months after the intervention, compared to the control group.
2. Our secondary hypotheses are that children who received the SRL intervention will see mid- to long-term improvements in
  - Standardised achievement testing scores as measured by the National Assessment Program in Literacy and Numeracy (NAPLAN),
  - Attendance and behavioural problems based on administrative records, and
  - Various wellbeing domains as measured by the Wellbeing and Engagement Collection (WEC)
    when compared to the control group.

For all hypotheses, we will compare heterogeneity in treatment effects by grade level and socio-economic position.

## Methods: participants, interventions and outcomes

### Trial design {8}

Pragmatic clustered by school with one class per grade level, parallel-group, randomised controlled superiority trial. Randomisation occurs at the level of the school to ensure no contamination across school grades (i.e. a school randomised to the intervention group will implement the intervention in grades 2, 4 and 6). Study participants will be consented for their data to be linked to administrative records allowing an additional population-wide matched parallel control arm. Our trial will follow the SPIRIT Guidelines [22] and relevant CONSORT statements [23, 24].

### Study setting {9}

The trial will be implemented across metropolitan, inner regional and rural areas of South Australia. Self-regulated learning is currently not a subject or a specified outcome within the Australian curriculum.

### Eligibility criteria {10}

Schools that have at least 15 students within one class in grade 2, grade 4, and grade 6 will be included. Those South Australian Government Primary Schools who meet these criteria, will be invited to participate by the Department for Education. The intervention will be delivered in grades 2, 4, and 6. All students within the selected classes will participate in the study. There will be no exclusion to the trial at class or individual child level. The invitation to schools will make it clear that it is expected that as part of agreeing to take part in the trial that they will be required to take part in the annual Wellbeing and Engagement Collection (WEC).

### Who will take informed consent? {26a}

School Principals will consent to their schools taking part in the trial. As a pragmatic trial, schools will deliver the intervention in their classes just as they would any other class content. Consent at the student level will not be required. Data collection will be undertaken by the schools with support from the central Department for Education. This data will then be linked to other administrative holdings within the Department before extraction and de-identification. Data will be provided to the academic team in a deidentified format for independent analyses.

### Additional consent provisions for collection and use of participant data and biological specimens {26b}

N/a as no biological specimens are collected.

## Interventions

### Explanation for the choice of comparators {6b}

In the control condition, teachers will continue their teaching practice as normal—i.e. standard practice.

### Intervention description {11a}

The intervention will be based on the Schunk et al. (2022) study. The intervention consists of a set of lessons introducing the process of Mental Contrasting with Implementation Intentions (MCII). MCII is a self-regulation technique that combines two cognitive strategies to help individuals achieve their goals [25]. The first strategy, mental contrasting, involves contrasting a desired future with the present reality to identify any potential obstacles that may hinder progress towards the goal. The second strategy, implementation intentions, involves creating specific plans that outline how and when the individual will take action towards the goal. Together, these two strategies can help individuals develop a clearer understanding of their goals and the steps needed to achieve them. By mentally contrasting the future and identifying obstacles, individuals can prepare for potential challenges and develop effective implementation intentions to overcome them. The MCII approach has been shown to be effective in various domains, including health behaviour change, academic achievement, and goal attainment [3, 26]. The learning lessons are directly tied to the teaching of skills that are fundamentally important for school children including practising reading and monitoring their own mistakes.

The SRL intervention will be adjusted to the Australian school system. Key differences between the original programme and the Australian version will be because of context, including a larger more diverse student population from suburban, regional and rural areas and a wider age range. This will result in pedagogical differences with the Australian version being more student focussed, allowing for greater differentiation. It is composed of a comprehensive suite of educational resources designed to scaffold student cognition, along with learning session plans spanning approximately five hours. These sessions will be spread over a consecutive five-week period, adhering to a pre-established schedule, and facilitated by the teachers. Based on the MCII strategy, the learning sessions follow the WOOPS structure and explore mistake detection in literacy and numeracy, followed by reading goals and lastly student-led goals. In general, the intervention will be given in the second lesson of the day to maximise student attention levels. Teachers will undertake a mandatory training session of at least 5 h that includes both the research background in self-regulated learning and the content of the learning sessions. Training for teachers will be conducted by a university (author SF) and department staff member. As a pragmatic trial, post the initial training, teachers will be left to implement the program as they see fit, with no observation or fidelity checks during implementation. It is worth noting, that in South Australia, schools and teachers are provided considerable agency and autonomy in the way they work towards curriculum outcomes. Post implementation, at the end of the school year (approximately 6 months post-intervention implementation), teachers will be asked to complete a survey aimed at determining their adherence to the learning sessions, how they implemented them (i.e. in a single lesson per week over 5 weeks or otherwise) and if they continued to imbed any of the SRL strategies into their everyday teaching practices.

### Criteria for discontinuing or modifying allocated interventions {11b}

There will be no criteria for discontinuing or modifying the intervention. Being a pragmatic trial, it is understood that not all students will receive the full “dose” of the intervention should they be absent on the day that a lesson was taught. In all circumstances, dose and implementation fidelity will be recorded.

### Strategies to improve adherence to interventions {11c}

All teachers within the intervention arm will receive standardised training. Scaffolding resources (posters, booklets, videos) will be standardised. Training attendance will be required prior to delivering the intervention.

### Relevant concomitant care permitted or prohibited during the trial {11d}

We are aware of an existing support program provided by the Department for Education aimed at providing professional development to school staff. This program primarily provides generalist information regarding self-regulation within a therapeutic framework, rather than a focus on self-regulated learning. The professional development is provided at the school level by occupational therapists and given staffing levels they operate under a waitlist. Their 2024 waitlist will be matched with our sample prior to randomisation and stratified accordingly.

### Provisions for post-trial care {30}

There will be no provisions for post-trial care.

### Outcomes {12}

#### Primary outcome

The primary outcome will be the student’s Self-Regulated Learning at 6 months post-implementation of the intervention, as assessed by the student’s teacher using a brief rating scale consisting of 14 items.

#### Secondary outcomes

Self-regulated learning will also be measured by the brief teacher-rated scale at 6 weeks and a student self-report measure with the same items will be utilised for grades 4 and 6. The student instrument will be implemented at 6 weeks, 6 months and 12 months post the intervention. Standardised academic achievement tests, Wellbeing and Engagement Collection (WEC) domains [27], school engagement, attendance and behaviour data will be obtained from the Department for Education through administrative data linkage.

#### Longer-term secondary outcomes

Academic and wellbeing measures will be obtained through administrative data linkage by the Department for Education. This will include the National Assessment Program for Literacy and Numeracy (NAPLAN) [28] undertaken for all students in grades 3, 5, 7 and 9 and the Wellbeing and Engagement Collection which is undertaken annually for all students from grade 4 through to grade 12. Data can be linked longitudinally at an individual level.

#### Covariates

Covariates will be obtained through linked education administrative data and will include the geographical socio-economic status of the primary residence of the child, parent's highest level of education, measures of reading abilities (as an indicator of pre-intervention cognition), disability status, school card status (poverty indicator), and prior school attendance records.

#### Exposure variable for per protocol analyses

Primary analyses will be intent-to-treat; however, student attendance for all sessions of the intervention will be obtained from departmental records. As such, we will undertake an additional per-protocol analysis where a student will be defined as receiving the intervention if they attended all sessions.

#### Costing data

Full cost details will be captured including upfront investment costs (e.g. resources, infrastructure, technical support, pre-service training) and recurrent costs (e.g. professional development, administrative support).

### Participant timeline {13}

Socialisation of the trial will be undertaken from November 2023, with school recruitment occurring at the same time. In the third school term, all intervention resources will have been fully adapted to the Australian context and modified to be appropriate for the three different grade levels.

Each of the four school terms is approximately 10 weeks. The teacher training will ideally be implemented late in the first term. The intervention will commence in all treatment schools across the three different grade levels in term 2 2024 (commences 29th April). We will provide video-based tutorials prior to each lesson of the intervention to remind teachers about the specific contents. Ideally, the intervention will be delivered from weeks 3 to 9 in terms 2, with flexibility given due to teacher sickness or other unforeseen disruptions.

**Fig. 1:**
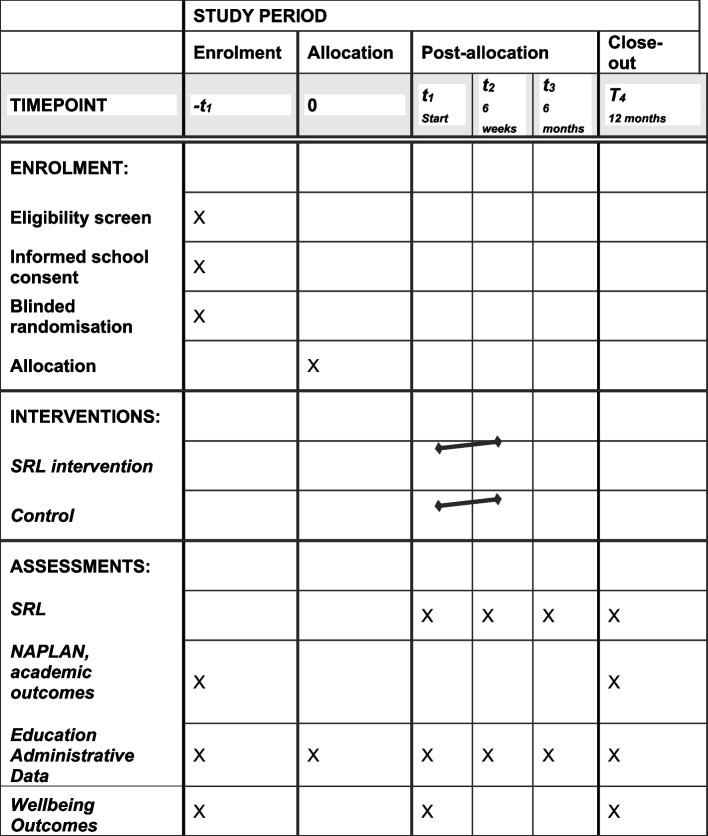
Schedule of enrolment, interventions, and assessments*

Limited trial-specific measurements will be collected pre and post-intervention to ensure as low a burden on the school system as possible. Where possible, all data pertinent to the trial will be collected via administrative data collection. Depending on the assessment these are collected at different times of the year and for different grade levels. Trial participants will be linked to the Department’s administrative data systems with a longitudinal data set linked and generated for analyses. Extractions from the Department systems will occur once per term post-completion of the intervention, with impact determined on the different outcomes as the students age into and thus complete the various outcomes of interest (Fig. 1).

### Sample size {14}

A sample of (at least) 56 schools will be required with at least one class in grade 2, grade 4 and grade 6, with an average cluster size of 19 children/class to detect a minimum impact of 0.25 SD effect size on the primary outcome with (at least) 80% power while accounting for the clustered design with an intraclass correlation coefficient (ICC) of 0.05. This results in a total sample of (at least) 56 clusters with an estimated 3192 students (or 1064/year level).

### Recruitment {15}

The Chief Executive of the Department for Education will invite schools to participate in the study. The department will facilitate the communication, departmental liaison and engagement with schools. Schools fulfilling the eligibility requirements can actively sign up for the study and will subsequently be randomised into treatment and control groups. Within each primary school a grade 2, 4, and 6 class will be randomly selected from the school class lists. Students in the classes will all receive the intervention in Term 2, 2024. Term 2 was selected as it is a “quieter” term, with the students more settled in class. This time frame also allows for a full 12 months before the primary academic outcomes are collected (NAPLAN). Given the uncontentious nature of the study, parental and student passive/opt-out consent will be employed for the trial.

## Assignment of interventions: allocation

### Sequence generation {16a}

Schools will be the level of randomisation and class the level of implementation. Randomisation will be stratified by socio-economic status blocks (using the Socio-Economic Indexes for Areas (SEIFA) indicator [29]) and metropolitan vs. non-metropolitan areas. The allocation sequence will be generated using computer-generated random numbers in Stata. Specifically, three block randomisation lists, one for each SES stratum will be generated with block sizes of 4 to ensure balanced allocation within each stratum. The advantage of numerous classes within the one school grade level has limited advantage for sample power due to the design effect associated with clustering. As such, at least one class per eligible grade level will be randomly selected for the intervention. Teachers will implement the intervention at the class level. The trial will be powered to assess the impact of the SRL intervention on three grades independently: grade 2 (early primary), grade 4 (mid primary), and grade 6 (late primary). The intervention will be implemented in literacy/reading lessons. Class lists will be provided by each of the participating schools for the process of randomisation. Note that schools in the treatment group may decide to implement the SRL intervention in more than one class within the school; however, only selected classes will be enlisted for the study. Schools in the control group will be required to not use the intervention (not even for non-enlisted classes).

We note that more than the required sample of 56 schools may wish to participate. As such we consider 56 as the minimum sample size, however the resultant study may be larger. Additionally, schools not meeting the eligibility criteria may still wish to participate in the study, for example, rural schools with multigrade classes. For such schools, we will include them as additional samples and block randomise accordingly.

### Concealment mechanism {16b}

Allocation will be concealed to prevent selection bias. Through a process of centralised allocation, schools will be recruited by Department for Education staff before allocation is determined. Once recruitment is complete, the allocation sequence will be generated by university staff using a project-specific school ID provided by the Department. University staff conducting the allocation will be blinded to school names to maintain impartiality. After allocation, the Department will match the project-specific school IDs to school names and subsequently inform the recruited schools of their assigned group (intervention or control). This process ensures that allocation remains concealed until assignments are finalised and communicated to the schools.

### Implementation {16c}

Randomisation will be undertaken by an independent member of Education Futures at the University of South Australia blinded to the school (cluster) names. All schools selected for participation will be randomised. Blinding at the student level will not be possible due to the pragmatic clustered design.

## Assignment of interventions: blinding

### Who will be blinded {17a}

Although random allocation will be undertaken blinded to school names or identifying details, post randomisation the study implementation cannot be blinded due to the clustered design. Outcome assessment will be undertaken by the Department and also not blinded. Analyses will be undertaken independently of the Department and the data analysts will be blinded to the allocation arm.

### Procedure for unblinding if needed {17b}

Participants are unblinded. Analysts will be blinded and unblinding will not be permissible for them.

## Data collection and management

### Plans for assessment and collection of outcomes {18a}

Assessment of self-regulated learning will be undertaken by the teacher of participants in grades 2, 4 and 6. For grades 4 and 6, the self-regulated learning assessment will additionally be self-completed by the student.

For all secondary outcomes (WEC and NAPLAN), data will be obtained from the Department for Education, South Australia. The WEC and NAPLAN are administered annually by the Department as part of standard practice. NAPLAN is typically conducted during the second full week of May each year in Australian schools. It is a nationwide assessment program used to measure the literacy and numeracy skills of students in grades 3, 5, 7, and 9. The exact timing of the Wellbeing and Engagement Collection (WEC) collection can vary from year to year, but generally takes place during the second term (April to July) of the school year. The WEC is administered to all schools that have agreed to participate (approximately 90% of all South Australian government schools). For the schools that do participate all students from grade 4 through to 12 will be requested to complete the survey.

### Plans to promote participant retention and complete follow-up {18b}

A requirement of the school recruitment will include a commitment to facilitate all data collection for the purposes of the RCT and to participate in the NAPLAN and WEC data collections. All students have a unique Education ID that transfers with them should they change schools. As such participating students will be tracked through the education system with data linked and made available to researchers.

The greatest risk to long-term follow-up data will be for the grade 6 students who in year 7 will transition from primary to high school. Approximately 10% of students transfer to the private education sector during this transition and will likely be lost to longer-term follow-up. It is not expected that this loss would differ across the control and intervention groups.

### Data management {19}

Much of the data will be collected via administrative data linkage on the basis of the student’s Education ID. Where trial-specific data collection is required, all will be conducted by trained assessors, whom have experience working in school settings. Assessors will have at least a bachelor’s level education. Data collection will be overseen by the Study Coordinator, however, will be collected and linked by the Department for Education. Once extracted and provided to the University the research team will be responsible for cleaning, coding security and storage as consistent with the University’s and ethical requirements. All analyses will be undertaken by the research team, independent of the Department for Education.

### Confidentiality {27}

Student-level data will never be received by the research team in an identified format. Students will be uniquely identified by their Education ID, so will remain known to the Department, but not the research team. Similarly, schools will be uniquely identified in all records by their school ID.

### Plans for collection, laboratory evaluation and storage of biological specimens for genetic or molecular analysis in this trial/future use {33}

N/a as no biological specimens will be collected.

## Statistical methods

### Statistical methods for primary and secondary outcomes {20a}

The primary analysis will use the intent-to-treat principle. Baseline balance will be checked between trial arms to see whether randomisation was successful. Estimation of treatment effects will be obtained from a generalised linear mixed model (GLMM) to produce the mean difference in scores for SRL intervention versus control. The GLMM will include a random effect for a cluster, and a random effect for the child taking account of the repeated measure design, a fixed effect for time, and use an exchangeable correlation structure of the appropriate variance–covariance matrix. The same analytical approach will be applied to secondary outcomes of interest. Longitudinal data collected for school cohort participants will be linked using student Education IDs. Student household characteristics may serve as predictors of interest in adjusted analyses.

### Interim analyses {21b}

N/a; there will be no interim analyses.

### Methods for additional analyses (e.g. subgroup analyses) {20b}

#### Economic assessment

Attention to the cost-effectiveness of the SRL intervention is a crucial component of this trial. This evaluation will apply an ingredients approach to assessing program costs, cost per beneficiary, and cost per impact.

### Implementation evaluation

Implementation evaluations are critical to any consideration of scale-up post the trial by providing key insights on the challenges, in terms of replicability, coverage and sustainability. For example, implementing the intervention in multiple grade levels will help to determine any age-related impacts. The trial's implementation evaluation will capture information relating to facilitators and barriers for participation in, and delivery of, the SRL intervention, as well as fidelity in delivery. A teacher fidelity and “dosage” survey will be implemented to all intervention teachers at the end of the school year and focus groups with these same teachers will be held in the following school year to ensure that there is no Hawthorne effect risk. One-on-one interviews will be held with school leaders, also in the following school year. Questions will include those pertaining to the adequacy of the teacher training, perceptions of student impact, reflections on any equity issues (i.e. can all students engage in the SRL approach equally), was the SRL approach transferable across different contexts (academic, social and behavioural), further teacher supports required, intentions to continue to implement the SRL approaches, considerations for scaling up such as competing professional development priorities. This will help determine the conditions required to support programme expansion, replication, and sustainable scale-up.

### Methods in analysis to handle protocol non-adherence and any statistical methods to handle missing data {20c}

Data will initially be analysed as intention to treat. It is likely that some students may not be exposed to all class sessions, for example, due to sickness. As such, “dose” will be captured by student attendance records. Other potential variations in fidelity, such as teacher sickness, will also be recorded by schools. These records will be used to determine any differential effects by dose. Per-protocol analyses could be undertaken, should a large number of students within the intervention arm not participate, however, we do not expect this to be warranted.

Imputation methods will be utilised to estimate missing values. Sensitivity analyses will also be conducted to assess the impact of missing data on the study findings by examining the robustness of the results by varying assumptions about the missingness mechanism and imputation models.

### Plans to give access to the full protocol, participant-level data and statistical code {31c}

This study protocol will be published open access. A curated de-identified and anonymized participant-level data set will be available to researchers on request, including relevant derived variables and statistical code.

## Oversight and monitoring

### Composition of the coordinating centre and trial steering committee {5d}

The Trial Scientific Steering Committee will consist of all authors on this protocol paper. All authors will be responsible for the scientific conduct of the trial under the leadership of Brinkman. In addition, there will be a Trial Project Management Team which will oversee the operational day-to-day requirements of the trial. Also chaired by Brinkman, this team will include authors Fowler, Gabriel, Marrone, and Engelhardt along with a minimum of two additional staff from the Department of Education.

### Composition of the data monitoring committee, its role and reporting structure {21a}

A smaller data management team will be co-led by Lam and Brinkman and a Department representative from their Business Intelligence Unit who maintains all data assets held by the Ministry.

### Adverse event reporting and harms {22}

This RCT is considered low risk, being non-invasive, non-clinical and implemented by the student’s teachers in a controlled class environment. The trial is being implemented under pragmatic conditions. Teachers can cease implementation at any time should they consider the programme to be ineffectual or create any undue harm. Further, no adverse harms were reported in the original RCT conducted in Germany under similar conditions.

### Frequency and plans for auditing trial conduct {23}

The Trial Project Management Team will be primarily responsible for monitoring implementation and fidelity. Administrative records will collect the attendance of students. Training attendance will be recorded and online training systems will record usage by class with downloads date and time stamped.

### Plans for communicating important protocol amendments to relevant parties (e.g. trial participants, ethical committees) {25}

Any changes to the research study protocol will be reported to the University of South Australia Human Research Ethics Committee and the South Australian Department for Education’s Research Evaluation Committee. The trial registration will also be updated.

### Dissemination plans {31a}

Results will be disseminated via academic publication to ensure peer review of the results and interpretation. Findings will additionally be widely disseminated within Australia to education and health policy audiences, participating schools and relevant professional associations (for example the Primary Principals Association of Australia). International dissemination will be achieved primarily by academic conferences and publications. Senior policy makers will be encouraged to disseminate the findings across their networks and this will be facilitated by the research team providing briefings and supporting targeted communication materials (PowerPoint slide deck, infographics, short video).

## Discussion

The study protocol presented in this paper outlines a pragmatic clustered randomised controlled trial (RCT) aimed at evaluating a self-regulated learning (SRL) intervention to be implemented in South Australian primary schools. The intervention is based on the Mental Contrasting with Implementation Intentions (MCII) approach, which combines cognitive strategies to help primary school students achieve their goals. The protocol seeks to replicate a similar trial conducted in Germany by Schunk et al. (2022) but adapts it to the Australian context and extends it to include grade levels 2, 4, and 6.

The rationale for conducting this trial lies in the importance of SRL skills in educational success and lifelong outcomes. However, there are gaps in the existing literature, such as the transferability of SRL skills across different subjects and different education systems and the lack of studies examining multiple student populations with consistent outcome measures. To date, few RCTs have been conducted in this area, especially with long-term follow-up and broad socioeconomic representation. This study protocol aims to address these gaps by evaluating the impact of the SRL intervention on multiple outcomes, across different grade levels, and for diverse student populations.

The primary objective of the trial is to adapt and implement the SRL intervention in Australian primary schools, assess its effects on self-regulated learning skills, and evaluate the intervention’s cost-effectiveness for potential scale-up in South Australia. Our primary outcome is a teacher-reported assessment of student’s self-regulated learning skills at 6 months after the intervention’s implementation. We selected this as the primary outcome measure, as it represents the earliest indication of the intervention’s impact. This measure is crucial for establishing whether any subsequent improvements in academic outcomes are attributable to enhanced self-regulated learning, rather than direct improvements in literacy and reading instruction. This assessment will be conducted near the end of the school year, before students move into different classes with new teachers, making it the latest feasible time point for evaluation. We acknowledge that teachers are not blinded to the trial arm due to the study design, which may introduce potential reporter bias. However, alternative methods, such as independent student observation or task-based assessments, were not considered to be feasible given the large sample and concerns from the Department for Education regarding the additional burden on teachers and students. The trial will also examine secondary outcomes, including academic achievement, attendance, behavioural problems, and various well-being domains. The study will involve a sample of at least 56 schools, with an average cluster size of 19 children per class, resulting in a total sample size of approximately 3192 students across three grade levels. Data will be collected through teacher-rated scales, administrative data linkage to educational outcomes, and the Wellbeing and Engagement Collection. Covariates, such as socioeconomic status and reading abilities, will also be considered in the analyses.

South Australia has advanced existing data linkage infrastructure, allowing for study participants to be identified within the population-wide linked datasets. A critique of RCTs, despite being the best study design to determine causal impact, is that they can lack external validity (and therefore generalisability) to the real-world population. By linking the study sample to the whole population data, we will quantify the generalisability and estimate the trial effects for the entire South Australian school population. We will also use the administrative linked data to quantify “spill-over” effects. This refers to situations where teachers who have been part of the intervention may teach other teachers their new knowledge, skills, and resources, thereby impacting other students in the same primary school who were not originally enrolled in the trial.

Linking the study participants to the population-wide data will also allow for an opportunity to determine the impact on outcomes post-schooling, such as mental health, criminal justice, and income, for example. Robust cost–benefit economic analyses will be conducted based on the actual rather than estimated long-term outcomes.

The trial will collect a range of process information, including data on the delivery of the intervention, the characteristics of the participants, and contextual factors that may affect implementation and outcomes. This information will help identify potential barriers and facilitators to intervention implementation, as well as factors that may influence intervention effectiveness in different population groups, in order to investigate aspects of equity.

This trial will provide valuable information on the acceptability and feasibility of the SRL intervention, including the experiences of participants and implementers, as well as the practical considerations of delivering the SRL intervention in the classroom for different grade levels. Overall, the process information collected will help identify the mechanisms of action of the SRL intervention, refine implementation strategies, and inform the translation of research findings into real-world practice, ultimately improving the quality and effectiveness of teaching strategies.

The engagement of members from various units within the Department for Education, as well as the fact that the intervention will be implemented by class teachers, should promote buy-in from stakeholders and bridge the gap between research and practice. The trial will be implemented under the auspices of the Fraser Mustard Centre agreement between the Department for Education and the University of South Australia. The aim of the Centre is to improve and promote the health and wellbeing of all children and young people in South Australia through the unique application of multidisciplinary research, while building capacity among public sector staff and academic researchers to design, undertake, and use research within the system. Consistent with the Centre's aims, trial results will be disseminated widely within the Department to policy and decision makers. If the trial yields positive results, the strong partnership between the Department and the University should increase the likelihood of sustained scale-up.

In conclusion, this study protocol outlines a rigorous research design to evaluate the implementation and effects of a self-regulated learning intervention in South Australian primary schools. By addressing the gaps in the existing literature and considering various outcomes and populations, this trial aims to provide valuable insights into the effectiveness and potential scalability of SRL interventions, ultimately contributing to educational policy and practice.

## Trial status

Protocol version 1.0. 20th July 2023. Recruitment began on the 1st of December 2023 and will be completed by April 12th 2024.

## Data Availability

The final trial dataset will be available to all academic authors. There are no contractual limitations on access, noting that the Department will provide de-identified data to the researchers.
